# Proteomic profiling of circulating plasma exosomes reveals novel biomarkers of Alzheimer’s disease

**DOI:** 10.1186/s13195-022-01133-1

**Published:** 2022-12-05

**Authors:** Huimin Cai, Yana Pang, Qi Wang, Wei Qin, Cuibai Wei, Ying Li, Tingting Li, Fangyu Li, Qigeng Wang, Yan Li, Yiping Wei, Longfei Jia

**Affiliations:** Innovation Center for Neurological Disorders and Department of Neurology, Xuanwu Hospital, Capital Medical University, National Clinical Research Center for Geriatric Diseases, 45 Changchun St, Beijing, 100000 China

**Keywords:** Alzheimer’s disease, Exosome, Proteomics, Biomarker, Diagnosis

## Abstract

**Background:**

Neuronal- and astrocyte-derived exosomes have been identified as an optimal source for screening biomarkers for Alzheimer’s disease (AD). However, few studies focus on the bulk exosome population isolated from plasma of AD. This study investigated whether proteins in bulk exosomes can aid in the diagnosis of AD.

**Methods:**

The plasma exosomes were collected by ultracentrifuge. Protein samples were extracted from exosomes. Cerebrospinal fluid levels of amyloid β (Aβ)42 and phosphorylated tau (P-tau)181 were measured for diagnostic purposes. A pilot study (controls, 20; AD, 20) followed by a second dataset (controls, 56; AD, 58) was used to establish a diagnostic model of AD. Mass spectrometry-based proteomics was performed to profile the plasma exosomal proteome. Parallel reaction monitoring was used to further confirm the differentially expressed proteins.

**Results:**

In total, 328 proteins in plasma exosomes were quantified. Among them, 31 proteins were altered in AD patients, and 12 were validated. The receiver operating characteristic curve analysis revealed a combination of six proteins (upregulated: Ig-like domain-containing protein (A0A0G2JRQ6), complement C1q subcomponent subunit C (C1QC), complement component C9 (CO9), platelet glycoprotein Ib beta chain (GP1BB), Ras suppressor protein 1 (RSU1); downregulated: disintegrin and metalloproteinase domain 10 (ADA10)) has the capacity to differentiate AD patients from healthy controls with high accuracy. Linear correlation analysis showed that the combination was significantly correlated with cognitive performance.

**Conclusions:**

The combination of plasma exosomal proteins A0A0G2JRQ6, C1QC, CO9, GP1BB, RSU1, and ADA10 acts as a novel candidate biomarker to differentiate AD patients from healthy individuals.

**Supplementary Information:**

The online version contains supplementary material available at 10.1186/s13195-022-01133-1.

## Background

Alzheimer’s disease (AD) is the most prevalent type of dementia in the elderly population, affecting nearly 50 million individuals worldwide [1]. AD is a debilitatingly progressive disease that creates considerable health, economic, and social issues [2]. Despite its great effect, accurate and timely diagnosis of AD remains challenging. The National Institute on Aging and Alzheimer’s Association (NIA-AA) [3] Research Framework proposed a biological definition of AD and addressed the role of biomarkers in AD diagnosis. Advances on biomarkers reflecting underlying pathophysiology include positron emission tomography (PET) and cerebrospinal fluid (CSF) analysis. While these biomarkers have improved diagnosis accuracy, they are limited in current clinical settings due to their high costs, inadequate availability, and invasive nature. As such, blood-based biomarkers, which are cost-effective, easily accessible, and less invasive, are of significant interest.

Exosomes are nanoscale extracellular vesicles released from all types of cells and widespread in nearly all body fluids. Exosomes contain proteins, nucleic acids, and other cellular components and play a critical role in intercellular communication [4]. Since exosomes and their constituents are involved in various physiological and pathological processes in the nervous system including neuroinflammation, synaptic plasticity, and dissemination of pathological molecules, they are considered ideal candidates to study the pathogenesis of AD and promising biomarkers to early diagnose and predict the progress of AD [5]. We and others used plasma neuronal-derived exosomes and revealed that amyloid β (Aβ)42, total tau (T-tau), and phosphorylated tau (P-tau)181 as well as other contents could differentiate AD patients from healthy individuals [6–9]. Furthermore, our previous work demonstrated that synaptic proteins (GAP43, neurogranin, SNAP25, and synaptotagmin 1) in plasma neuronal-derived exosomes served as effective biomarkers to predict AD 5 to 7 years before the clinical onset [10]. Similarly, alteration of various contents in plasma astrocyte-derived exosomes has also been reported in AD patients [11, 12]. These findings support that exosomes from nerve cells, such as neurons and astrocytes, can serve as a promising resource for screening biomarkers for AD. However, few studies focus on bulk exosome population isolated from plasma of AD. As collection of exosomes generated from neurons and astrocytes is expensive and time-consuming, seeking biomarkers in bulk exosomes may provide a convenient and low-cost approach for further clinical translation. In addition, increasing evidence showed that AD may be considered a systemic syndrome with multiple peripheral organs involved [13]. In this context, seeking altered proteins from bulk exosomes in plasma is needed to further understand AD pathogenesis.

Herein, we proposed an unbiased proteomics analysis of circulating plasma exosomes in AD patients and healthy controls for a comprehensive search for novel diagnostic biomarkers. The study aimed to explore the proteomic profiling of circulating plasma exosomes in AD patients and healthy controls and determine their diagnostic capacity.

## Methods

### Subjects

The Institutional Review Board of Xuanwu Hospital, Capital Medical University, approved the following protocol and written informed consent was obtained from all participants or their legal representatives before enrollment. In total, 154 subjects based on two datasets were included. Dataset 1 was obtained from a Beijing center (*n* = 40; controls, 20; AD, 20); dataset 2 was collected from centers in the provinces of Shandong, Henan, and Guangxi (*n* = 114, controls, 56; AD, 58). All AD patients fulfilled the 2011 criteria of the NIA-AA for probable AD dementia [3]. A CSF level of P-tau/Aβ42 > 0.14 was employed as fluid biomarkers to distinguish AD patients from healthy controls, which was calculated based on our previously published data [6] and is consistent with other studies’ findings [14]. We additionally included the CSF Aβ42 cutoff of 500 pg/ml as another diagnostic criterion since low CSF Aβ42 is the critical pathological change in AD according to the amyloid, tau, and neurodegeneration (ATN) framework [15].

### Collection of exosomes from blood samples by ultracentrifuge

Exosomes were isolated according to a published protocol with minor modifications [16]. Briefly, 5 mL of plasma sample were diluted with 30 mL PBS and centrifuged at 500 × g for 30 min followed by 2000 × g for 30 min at 4°C to remove cell debris. The supernates were centrifuged at 100,000 × g for 1.5 h at 4°C. The supernates were collected as negative control, and the pellets were washed with 10 mL PBS and then filtered using VacuCap 60 Devices (0.1 μm, Pall Life Sciences, Washington, NY, USA). The pellets were resuspended with 100 ml PBS and stored at − 80°C.

### Confirmation of exosomal collection

Transmission electron microscopy (TEM) and Western blot were conducted to confirm the successful collection of plasma exosomes following a standardized protocol as previously described [6]. Anti-Alix antibody was used in Western blot to identify exosomal marker (1:1000, Cell Signaling Technology, Danvers, MA, USA). Exosomes were measured using centrifuged samples, with supernates serving as negative control.

### CSF collection

CSF samples were collected and handled following the international standardized protocol [17]. Immediately after blood collection, lumbar punctures were performed to obtain 15 mL of CSF with 20-gauge atraumatic needles. Within 2 h, CSF samples were centrifuged at 2000 × g for 10 min at room temperature and kept at − 80°C in polypropylene tubes until further analysis.

### Protein measurements

Enzyme-linked immunosorbent assay (ELISA) was used to quantify the CSF levels of Aβ42, T-tau, and P-tau181, as well as the level of exosomal markers: CD9, CD63, and CD81. Details of the ELISA kits used in this research are described in Additional file 1: Table S1. All results were within the detection limit of the ELISA kits. All measurements were performed by a technician who was blinded to the clinical data.

### Proteome analysis

Proteomics was performed with liquid chromatography coupled to tandem mass spectrometry (LC-MS/MS) analysis and validated by targeted proteomic analysis using parallel reaction monitoring (PRM) according to Additional file 1:materials and methods.

### Statistical analysis

Data were analyzed using SPSS v.22 (IBM Corp, Armonk, NY, USA) and Stata 13.0 (StataCorp LLC, College Station, TX, USA). Datasets 1 and 2 were analyzed separately. Baseline characteristics and concentrations of biomarkers between groups were compared by *χ*^2^ tests for categorical data and Welch’s *t*-tests or analyses of variance (ANOVAs) for continuous data, as appropriate. To identify the differentially expressed proteins, the *P* values were corrected by false discovery rate (FDR). In dataset 2, the predicted values were generated by a binary logistic regression model using age, sex, education years, and *APOE* ε4 status as covariates, and then examined by receiver operating characteristic (ROC) curve analysis. Tolerances, variance inflation factors (VIFs), eigenvalues, and condition indices were used to calculate the multicollinearity between each protein. All tests were two-tailed, with *P* < 0.05 set as the level of statistical significance.

## Results

### Subject characteristics

Of 40 participants in dataset 1, mean age was 67.5(SD, 5.4) years and 20 (50%) were women. Of 114 participants in dataset 2, mean age was 68.7 (SD, 6.5) years, and 59 (51.8%) were women. The demographic and clinical characteristics of the study participants are shown in Tables 1 and 2. There was no significant difference between groups in both datasets with respect to age, sex, or education years. The levels of Aβ42 (*P* < 0.001, for both datasets) , T-tau (*P* < 0.001, for both datasets), P-tau181 (dataset 1: *P* = 0.001; dataset 2: *P* < 0.001), the *APOE* ε4 status (dataset 1: *P* = 0.03; dataset 2: *P* < 0.001), and Mini-Mental State Examination (MMSE) scores (*P* < 0.001, for both datasets) were as expected significantly different between groups in both datasets as shown in Tables 1 and 2.

**Table 1:** Characteristics of participants in dataset 1

Characteristic	Total sample (*n* = 40)	Controls (*n* = 20)	AD (*n* =20)	*P* value
Age, mean (SD)	67.5 (5.4)	66.8 (5.8)	68.2 (5.0)	0.43
Education year, mean (SD)	9.1 (2.6)	9.9 (3.0)	8.4 (1.9)	0.07
Women, no. (%)	20 (50.0)	10 (50.0)	10 (50.0)	1.00
*APOE* ε4 positive (%)	12 (30.0)	4 (20.0)	8 (40.0) *	0.03
MMSE score (SD)	23.9 (5.2)	28.9 (0.6)	19.0 (1.8) *	< 0.001
Aβ42 (pg/ml), mean (SD)	565.3 (250)	762.1 (175.5)	368.5 (126.4) *	< 0.001
P-tau181 (pg/ml), mean (SD)	86.2 (53.7)	58.6 (26.4)	113.8 (60.1) *	0.001
P-tau181/Aβ42, mean (SD)	0.20 (0.2)	0.08 (0.03)	0.32 (0.14) *	< 0.001
T-tau (pg/ml), mean (SD)	465.8 (185.8)	335.2 (89.3)	596.4 (179.0) *	< 0.001

The values of age, education years, and MMSE are shown as mean (standard deviation)

*Abbreviations*: *AD*, Alzheimer’s disease; *Aβ*, amyloid β; *APOE ε4*, apolipoprotein ε4; *MMSE*, Mini-Mental State Examination; *P-tau*, phosphorylated tau; *SD*, standard deviation; *T-tau*, total tau

*P < 0.05 compared to controls

**Table 2:** Characteristics of participants in dataset 2

Characteristic	Total sample (*n* = 114)	Controls (*n* = 56)	AD (*n* =58)	*P* value
Age, mean (SD)	68.7 (6.5)	68.3 (6.6)	69.1 (6.4)	0.50
Education year, mean (SD)	8.9 (2.2)	9.1 (2.3)	8.7 (2.1)	0.94
Women, no. (%)	59 (51.8)	29 (51.8)	30 (51.7)	1.00
*APOE* ε4 positive (%)	34 (29.8)	10 (17.9)	24 (41.4) *	< 0.001
MMSE score (SD)	24 (5.2)	28.9 (0.7)	19.2 (2.4) *	< 0.001
Aβ42 (pg/ml), mean (SD)	526.6 (215.8)	702.3 (149.5)	357 (105.8) *	< 0.001
P-tau181 (pg/ml), mean (SD)	83.3 (56.1)	46 (24.8)	119.2 (54.4) *	< 0.001
P-tau181/Aβ42, mean (SD)	0.20 (0.16)	0.07 (0.03)	0.33 (0.11) *	< 0.001
T-tau (pg/ml), mean (SD)	496.5 (202.3)	317.2 (84.9)	616.6 (171.4) *	< 0.001

The values of age, education years, and MMSE are shown as mean (standard deviation)

*Abbreviations*: *AD*, Alzheimer’s disease; *Aβ*, amyloid β; *APOE ε4*, apolipoprotein ε4; *MMSE*, Mini-Mental State Examination; *P-tau*, phosphorylated tau; *SD*, standard deviation; *T-tau*, total tau

*P < 0.05 compared to controls

### Confirmation of exosomal collection

**Fig. 1:**
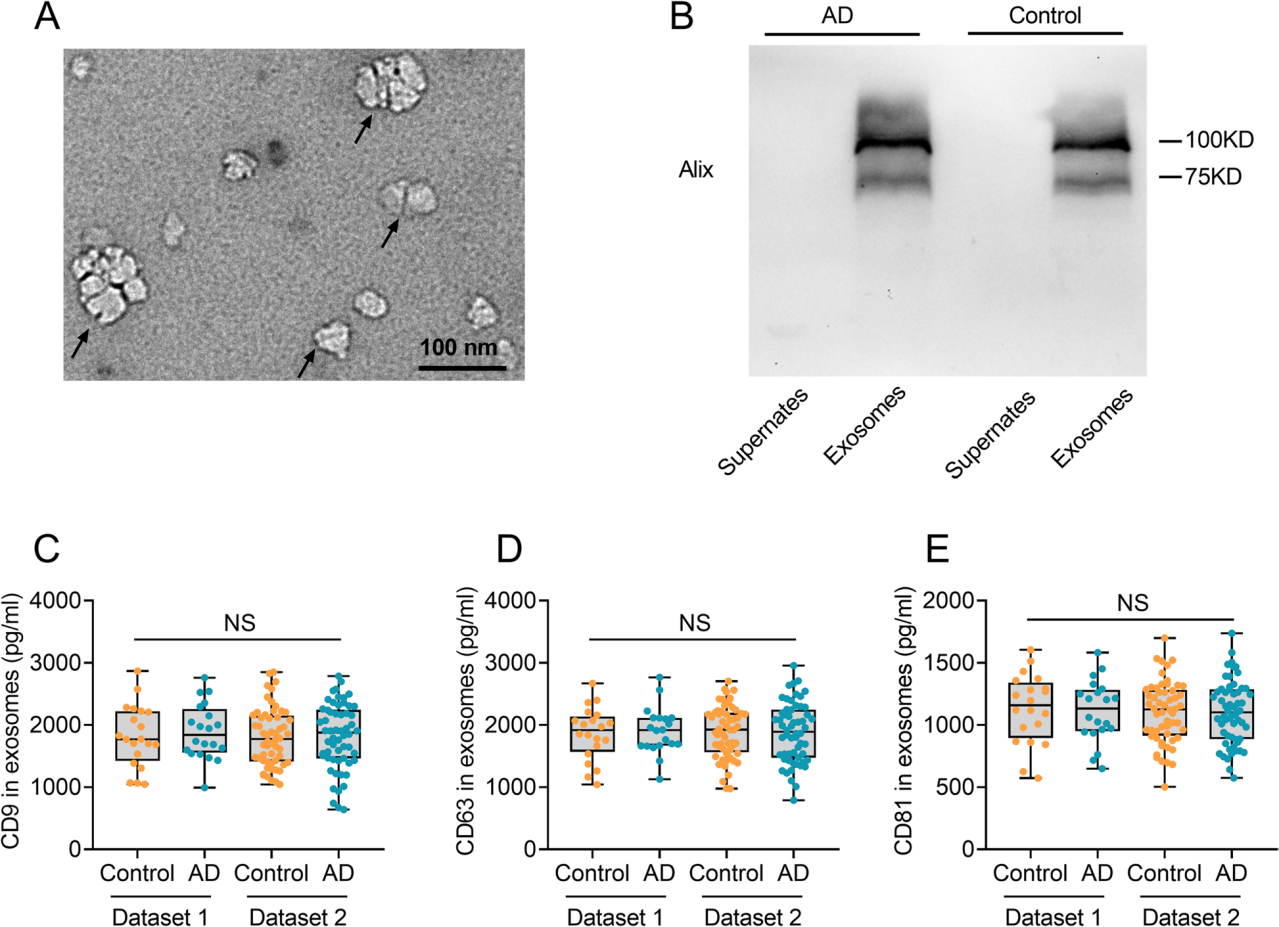
Confirmation of exosomes by transmission electron microscopy (TEM), Western blot, CD9, CD63, and CD81. **A** Plasma samples contained vesicles of typical exosomes morphology (black arrow) under TEM from an AD patient. Scale bar = 100 nm. **B** Western blot analysis of Alix, a common exosome surface marker, expression in the exosome samples. Supernates served as the negative control. **C**–**E** Measurement of CD9 (**C**), CD63 (**D**), and CD81 (**E**) in exosomes. AD, Alzheimer’s disease; NS, no significance

The exosome samples were identified by TEM. Representative TEM images of exosomes from an AD patient (Fig. 1A) and a healthy control (Additional file 2: Figure S1) are shown. Western blot analysis revealed that the exosome marker protein, Alix, was highly expressed in the exosome samples, while the supernates were negative for the marker (Fig. 1B). These findings suggested that the exosomes have been successfully collected from blood samples. The levels of CD9 (Fig. 1C), CD63 (Fig. 1D), and CD81 (Fig. 1E) in all samples were measured and no significant difference was detected between AD patients and controls.

### Detection of differentially expressed exosomal proteins

**Fig. 2:**
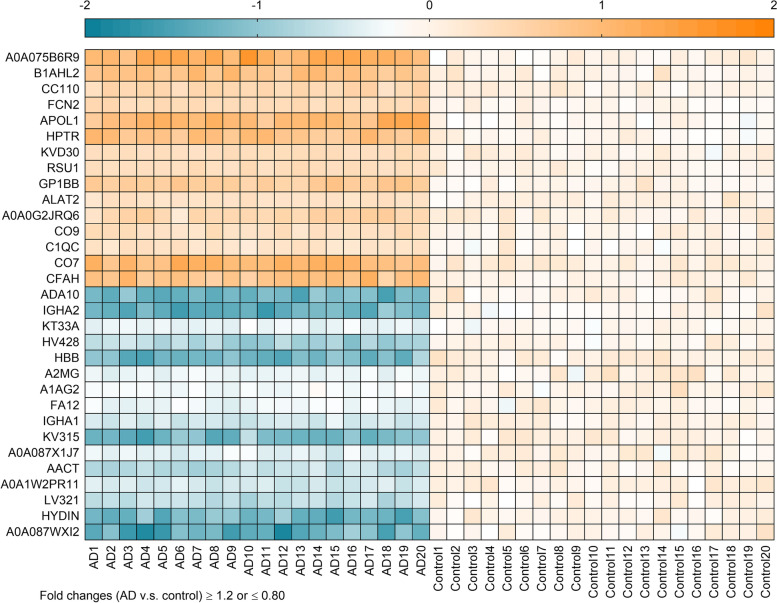
Heat map of 15 upregulated and 16 downregulated proteins in the pilot study (dataset 1). The heatmap was generated by log_2_ transformation of fold changes, with positive values representing upregulation (depicted in orange), and negative values representing downregulation (depicted in blue). AD, Alzheimer’s disease; A0A075B6R9, probable non-functional immunoglobulin kappa variable 2D-24; B1AHL2, fibulin-1; CC110, coiled-coil domain-containing protein 110; FCN2, ficolin-2;APOL1, apolipoprotein L1; HPTR, haptoglobin-related protein; KVD30, immunoglobulin kappa variable 2D-30; RSU1, Ras suppressor protein 1; GP1BB, platelet glycoprotein Ib beta chain; ALAT2, alanine aminotransferase 2; A0A0G2JRQ6, Ig-like domain-containing protein; CO9, complement component C9; C1QC, complement C1q subcomponent subunit C; CO7, complement component C7; CFAH, complement factor H; ADA10, disintegrin and metalloproteinase domain 10 (ADAM10); IGHA2, immunoglobulin heavy constant alpha 2; KT33A, keratin, type I cuticular Ha3-I; HV428, immunoglobulin heavy variable 4-28; HBB, hemoglobin subunit beta; A2MG, alpha-2-macroglobulin; A1AG2, alpha-1-acid glycoprotein 2; FA12, coagulation factor XII; IGHA1, immunoglobulin heavy constant alpha 1; KV315, immunoglobulin kappa variable 3-15; A0A087X1J7, glutathione peroxidase; AACT, alpha-1-antichymotrypsin; A0A1W2PR11, HLA class I histocompatibility antigen, C alpha chain; LV321, immunoglobulin lambda variable 3-21; HYDIN, hydrocephalus-inducing protein homolog; A0A087WXI2, IgGFc-binding protein

In a pilot study with a relatively small sample size (dataset 1), a total of 328 exosomal proteins were quantified in the blood of AD patients and controls. Among these proteins, 15 proteins were significantly upregulated and 16 were significantly downregulated in the AD group relative to the control group, as determined by fold changes ≥ 1.2 or ≤ 0.80. The expression levels of 31 proteins detected in the AD group compared to the control group are depicted in Fig. 2.

### Confirmation of differentially expressed exosomal proteins

**Fig. 3:**
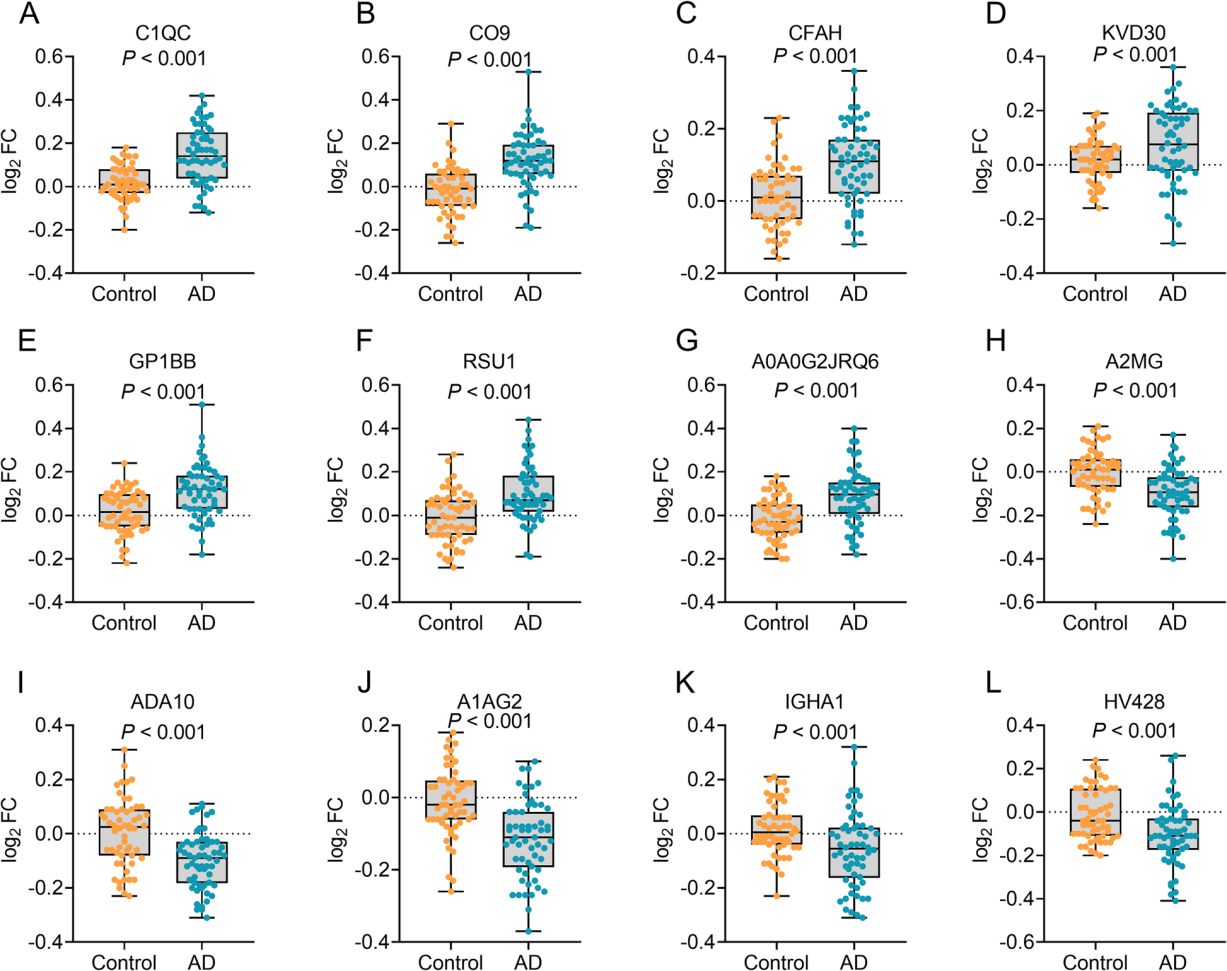
The measurements of exosomal proteins in dataset 2. C1QC (**A**), CO9 (**B**), CFAH (**C**), KVD30 (**D**), GP1BB (**E**), RSU1 (**F**), and A0A0G2JRQ6 (**G**) were increased in Alzheimer’s disease (AD) patients, and A2MG (**H**), ADA10 (**I**), A1AG2 (**J**), IGHA1 (**K**), and HV428 (**L**) were decreased in AD patients. AD, Alzheimer’s disease; FC, fold change. C1QC, complement C1q subcomponent subunit C; CO9, complement component C9; CFAH, complement factor H; KVD30, immunoglobulin kappa variable 2D-30; GP1BB, platelet glycoprotein Ib beta chain; RSU1, Ras suppressor protein 1; A0A0G2JRQ6, Ig-like domain-containing protein; A2MG, alpha-2-macroglobulin; ADA10, disintegrin and metalloproteinase domain 10 (ADAM10); A1AG2, alpha-1-acid glycoprotein 2; IGHA1, immunoglobulin heavy constant alpha 1; HV428, immunoglobulin heavy variable 4-28

A validation cohort with a larger sample size (dataset 2) was recruited to validate the 31 proteins differentially expressed between AD patients and controls. Seven upregulated (complement C1q subcomponent subunit C (C1QC), complement component C9 (CO9), complement factor H(CFAH), immunoglobulin kappa variable 2D-30 (KVD30), platelet glycoprotein Ib beta chain (GP1BB), Ras suppressor protein 1(RSU1), Ig-like domain-containing protein (A0A0G2JRQ6)) and five downregulated proteins (Alpha-2-macroglobulin (A2MG), disintegrin and metalloproteinase domain 10 (ADA10), alpha-1-acid glycoprotein 2 (A1AG2), immunoglobulin heavy constant alpha 1 (IGHA1), immunoglobulin heavy variable 4-28 (HV428)) from the pilot study were confirmed to be significantly differentially expressed in dataset 2 (Fig. 3). To obtain detailed biological information of the differentially expressed proteins, we performed bioinformatic analysis on the 12 proteins. GO analysis revealed that most of the proteins were involved in regulation of immune system and protein activation cascade (Additional file 3: Figure S2A, B) and functioned as binding molecules in different cellular processes (Additional file 3: Figure S2A, C). Most of the proteins were localized to the extracellular space and extracellular vesicles (Additional file 3: Figure S2A, D). KEGG pathway analysis showed that the proteins were most enriched in immune system and infectious disease related pathways, such as complement and coagulation cascades (Additional file 4: Figure S3A, B). Furthermore, B cell receptor signaling pathway as well as complement and coagulation cascades were most densely connected to enriched pathways (Additional file 4: Figure S3C). The enriched pathways were related to late-onset AD and cerebral amyloid angiopathy (Additional file 4: Figure S3D).

### Diagnostic panel of exosomal proteins

**Fig. 4:**
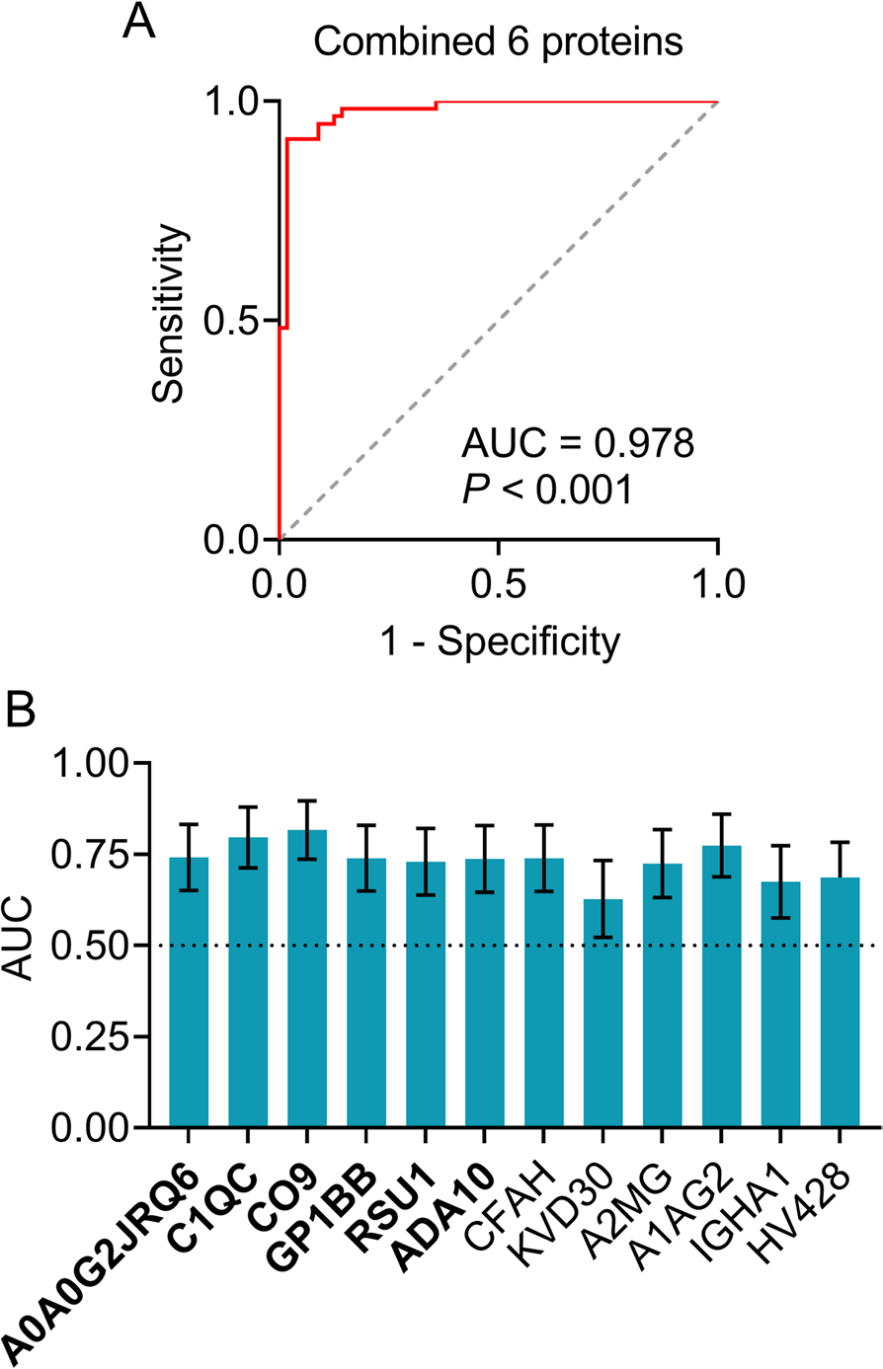
Establishment of diagnostic panel for Alzheimer’s disease. **A** Receiver operating characteristic (ROC) curve analyses of the six-protein panel. **B** ROC analyses of 12 individual exosomal protein. The six proteins included in the panel are in bold. AUC, area under the curve; A0A0G2JRQ6, Ig-like domain-containing protein; C1QC, complement C1q subcomponent subunit C; CO9, complement component C9; GP1BB, platelet glycoprotein Ib beta chain; RSU1, Ras suppressor protein 1; ADA10, disintegrin and metalloproteinase domain 10 (ADAM10); CFAH, complement factor H; KVD30, immunoglobulin kappa variable 2D-30; A2MG, alpha-2-macroglobulin; A1AG2, alpha-1-acid glycoprotein 2; IGHA1, immunoglobulin heavy constant alpha 1; HV428, immunoglobulin heavy variable 4-28. *P* < 0.001 (**A**) or 0.01 (**B**)

A logistic model was developed to estimate the performance of the aforementioned 12 proteins to distinguish AD patients from controls, using diagnosis (AD versus control) as the dependent variable and the 12 proteins as covariates. After adjusting for age, sex, education years, and *APOE* ε4 status, a panel of six proteins (upregulated: A0A0G2JRQ6, C1QC, CO9, GP1BB, and RSU1; downregulated: ADA10) was found to be associated with AD. Age, sex, and education years were removed from further analysis since they had a *P* value > 0.05 in the logistic model. Multicollinearity diagnostics among the six proteins in AD patients and controls revealed that all tolerances were > 0.1, VIFs were < 10, eigenvalues were > 0, and condition indices were < 30, indicating the absence of significant multicollinearity among the six proteins. To determine the diagnostic power of the six-protein panel, the predictive values of the panel from the logistic model were further evaluated by ROC curve analysis. The obtained results revealed that the six-protein panel displayed an excellent diagnostic power for AD with a significantly higher area under the curve (AUC = 0.978, *P* < 0.001, Fig. 4A) than those of the individual proteins (AUC = 0.627–0.774, Fig. 4B), indicating that a combination of the six proteins was necessary to obtain an effective diagnosis.

### Correlations of MMSE values with exosomal protein levels

To further investigate the relationships between exosomal protein levels and cognitive impairment in AD, linear correlation analyses were conducted between MMSE scores and the levels of A0A0G2JRQ6, C1QC, CO9, GP1BB, RSU1, and ADA10 in AD patients. Significant correlation was detected between the combined exosomal protein levels and MMSE scores in AD patients (adjust *R*^2^ = 0.563, *P* < 0.001, Additional file 1: Table S2), while only weak correlations were observed between individual single exosomal protein and MMSE scores (*R*^2^ = A0A0G2JRQ6, 0.193; C1QC, 0.207; CO9, 0.231; GP1BB, 0.176; RSU1, 0.148; ADA10, 0.125, all *P* < 0.001, Additional file 1: Table S2), implying the potentials of the six-protein panel to predict cognitive impairment.

## Discussion

In the current study, we analyzed two independent datasets to investigate proteomic profiling of circulating plasma exosomes in AD. We found that a panel of six exosomal proteins was associated with AD. To the best of our knowledge, this is the first time that a diagnostic model of AD was established using bulk exosomes of plasma. It is less invasive and antibody-independent and may therefore be extensively used for AD screening in the general population of old adults.

Biomarkers have important roles in AD diagnosis [3] and research [18]. The use of serum or plasma in the diagnosis of AD is gaining more and more attention as it has low invasiveness and relatively low cost. Increasing evidence supports multiple promising blood biomarkers, such as Aβ42 [19], neurofilament light protein [20], P-tau181, and P-tau217 [21]. In this study, we generated a diagnostic panel of AD by detecting exosomal proteins in the blood. Label-free proteomics revealed 15 upregulated and 16 downregulated exosomal proteins in AD. Twelve differentially expressed exosomal proteins were further confirmed by PRM, which is a targeted method for the identification and quantification of proteins due to its reproducible and consistent results [22] and has been extensively used in multiple studies [23, 24]. Among the 12 proteins, the combination of six proteins was strongly associated with AD and can distinguish AD from healthy controls with high performance. Furthermore, the six-protein panel is significantly positively correlated with cognitive performance, making it an effective predictor for cognitive decline. Our findings provided convenient and antibody free biomarkers for AD.

By proteomic analysis, we identified a six-protein panel in circulating exosomes that may be a novel biomarker for AD. The differentially expressed proteins are associated with several processes: inflammation and immunity, coagulation, and the proteolytic process of amyloid precursor protein (APP). The levels of several complement-related proteins were elevated in our study, which is consistent with earlier findings in astrocyte-derived exosome research [12]. Compelling evidence revealed that the complement system was essential in AD pathogenesis [25]. For example, C1QC associates with other subunits to yield C1q, the initiating component of the classical complement pathway. C1q tags the tau-affected synapses by opsonization and induces subsequent microglia phagocytosis [26]. Furthermore, mouse models showed that dysregulated C1q-mediated synaptic pruning and/or spine density loss was associated with behavioral deficits, implicating its detrimental role in the inflammatory process [27]. Nevertheless, C1q exerts direct protective effects on primary cultured neurons against amyloid-induced toxicity [28, 29]. Together, these findings indicate C1q plays dual roles in AD pathogenesis. Another complement, CO9, is a key subcomponent of membrane attack complex (MAC), which plays a significant role in the downstream cascade of complement-meditated pathways. Studies found that MAC co-localized with Aβ plaques and tau tangles in the brains of AD patients, suggesting that CO9 may contribute to AD pathogenesis [25]. GP1BB is a subunit of the GPIb-V-IX complex, which is a transmembrane protein in platelets and constitutes the receptor for von Willebrand factor (vWF). vWF is abundantly expressed in cerebrovascular endothelium and associated with enhanced inflammatory activity, promoting endothelial dysfunction and cerebral amyloid angiopathy pathology [30, 31]. Therefore, it is possible that upregulated GP1BB boosts intracellular signaling response to vWF and, ultimately, deteriorates neurotoxicity. To date, no physiological function of RSU1 has been reported in AD. Nonetheless, there exist two intriguing connections between RSU1 and AD pathogenesis. RSU1 inhibits c-Jun N-terminal kinase (JNK) and enhances extracellular signal-regulated kinase (ERK) activation [32]. JNK activation links to amyloidogenic protein processing, whereas JNK suppression leads to a significant reduction in Aβ42 peptide levels and total plaque burdens as well as to an increased number of neurons and enhanced cognition [33]. Moreover, the ERK pathway is involved in neuroprotection against oxidative stress and Aβ toxicity [34]. As a hypothesis, increased RSU1 level measured in AD patients might reflect a compensatory neuroprotection phenomenon in response to excess Aβ burden. The single downregulated exosomal protein ADA10, also known as disintegrin and metalloproteinase domain 10 (ADAM10), is the major α-secretase for APP processing. ADA10 has a role not only in reducing the production of Aβ peptides and relieving the pathologic impairment in AD but may also in reducing tau pathology, preserving synaptic functions, and promoting hippocampal neurogenesis [35]. In line with our study, ADA10 in the platelets has been proposed as a potential biomarker for early diagnosis of AD [36]. Taken together, our findings identify a panel of exosomal proteins as an accurate biomarker for diagnosis of AD and extend the understanding of the disease, albeit it remains undetermined how these proteins contribute to the disease.

### Limitations

The study has several limitations. First, this study was limited by its cross-sectional design. Although we confirmed that a panel of six exosomal proteins may be a promising biomarker for AD, longitudinal studies are more appropriate to evaluate the performance of these biomarkers. Second, although the study involved two independent datasets, the results should be validated in other datasets. Finally, this study did not include patients with mild cognitive impairment who developed AD or stable amnestic mild cognitive impairment. Therefore, it is not clear whether our method is suitable for predicting progression from prodromal to probable AD.

## Conclusions

In conclusion, our study indicates that a panel of six exosomal proteins is a potential blood biomarker for AD. The study brings more insight into understanding AD and indicates that proteomic profiling from circulating plasma exosomes may facilitate AD diagnosis, warranting further validation in independent cohorts.

## Supplementary Information

**Additional file 1: Table S1.** ELISA kits information. **Table S2.** The association between exosomal protein levels and MMSE scores in Dataset 2. Materials and methods.

**Additional file 2: Figures S1.** Confirmation of exosomes by transmission electron microscopy (TEM).

**Additional file 3: Figures S2.** GO enrichment analysis of the 12 differentially expressed proteins.

**Additional file 4: Figures S3.** KEGG pathway enrichment analysis of the 12 differentially expressed proteins.

## Data Availability

The datasets used and/or analyzed during the current study are available from the corresponding author on reasonable request.

## Abbreviations

A0A0G2JRQ6: Ig-like domain-containing protein
A1AG2: Alpha-1-acid glycoprotein 2
A2MG: Alpha-2-macroglobulin
Aβ: Amyloid β
AD: Alzheimer’s disease
ADA10: Disintegrin and metalloproteinase domain 10
ANOVAs: Analyses of variance
*APOE*: Apolipoprotein E
APP: Amyloid precursor protein
ATN: Amyloid, tau and neurodegeneration
AUC: Area under the curve
C1QC: Complement C1q subcomponent subunit C
CFAH: Complement factor H
CO9: Complement component C9
CSF: Cerebrospinal fluid
ELISA: Enzyme-linked immunosorbent assay
ERK: Extracellular signal-regulated kinase
FDR: False discovery rate
HV428: Immunoglobulin heavy variable 4-28
IGHA1: Immunoglobulin heavy constant alpha 1
JNK: c-Jun N-terminal kinase
KVD30: Immunoglobulin kappa variable 2D-30
LC-MS/ MS: Liquid chromatography coupled to tandem mass spectrometry
MAC: Membrane attack complex
MMSE: Mini-Mental State Examination
NIA-AA: National Institute on Aging and Alzheimer’s Association
PET: Positron emission tomography
PRM: Parallel reaction monitoring
P-tau: Phosphorylated tau
RSU1: Ras suppressor protein 1
TEM: Transmission electron microscopy
T-tau: Total tau
VIFs: Variance inflation factors
vWF: Von Willebrand factor
