# Supplementary material for: Proteomic profiling of circulating plasma exosomes reveals novel biomarkers of Alzheimer’s disease
Source: Alzheimers Res Ther. 2022 Dec 5;14:181. doi: 10.1186/s13195-022-01133-1 (PMC9720984; doi:10.1186/s13195-022-01133-1)
Supplement: Supplementary file 1 — Additional file 1: Table S1. ELISA kits information. Table S2. The association between exosomal protein levels and MMSE scores in Dataset 2. Materials and methods. [file 13195_2022_1133_MOESM1_ESM.docx]

**Supplementary Material**

**Title:**

**Proteomic profiling of circulating plasma exosomes reveals novel biomarkers of Alzheimer’s disease**

Huimin Cai, et al.

The Supplementary materials include the following information:

Supplementary Table 1. ELISA kits information

Supplementary Table 2. The association between exosomal protein levels and MMSE scores in Dataset 2

Supplementary materials and methods

**Supplementary Table 1. ELISA kits information**

| **ELISA kits** | **Catalog numbers** |
| --- | --- |
| **CD9** |  |
| LifeSpan BioSciences (USA) | LS-F6853 |
| **CD63** |  |
| RayBiotech (USA) | ELH-CD63-1 |
| **CD81** |  |
| LifeSpan BioSciences (USA) | LS-F7468-1 |
| **Aβ42** |  |
| INNOTEST (Japan） | 81576 |
| **P-tau181** |  |
| INNOTEST (Japan) | 81581 |
| **T-tau** |  |
| INNOTEST (Japan) | 81572 |

**Supplementary Table 2. The association between exosomal protein levels and MMSE scores in Dataset 2**

| Proteins | R^2^ | *P* value |
| --- | --- | --- |
| A0A0G2JRQ6 | 0.193 | < 0.001 |
| C1QC | 0.207 | < 0.001 |
| CO9 | 0.231 | < 0.001 |
| GP1BB | 0.176 | < 0.001 |
| RSU1 | 0.148 | < 0.001 |
| ADA10 | 0.125 | < 0.001 |
| Combination of six proteins | 0.563^a^ | < 0.001 |

Abbreviations: MMSE, Mini-Mental State Examination; A0A0G2JRQ6: Ig-like domain-containing protein; C1QC: Complement C1q subcomponent subunit C; CO9: Complement component C9; GP1BB: Platelet glycoprotein Ib beta chain; RSU1: Ras suppressor protein 1; ADA10: Disintegrin and metalloproteinase domain 10 (ADAM10). ^a^ adjusted R^2^.

**Supplementary materials and methods**

**Shotgun proteomics**

**Peptide Preparation**

Ten µg of exosomes protein samples were isolated under denatured conditions using 12% Tris-glycine gels. Then gel staining was performed at room temperature for 1 h in a Coomassie blue solution (20% methanol; 10% acetic acid; 0.1% Coomassie Brilliant Blue R). Gel destaining was subsequently performed at room temperature for 2 h in a destaining solution (50% methanol; 10% acetic acid). Each lane of the gels was cut into pieces of 1 mm^3^. Gel pieces were moved into a 1.5 ml tube, and destained twice using 50% ethanol in 50 mM ammonium bicarbonate at 22℃ for 15min and dehydrated with 100% acetonitrile for 5 min. Dehydrated gel pieces were reduced with 10 mM dithiothreitol at 56℃ for 30 min. The dithiothreitol solution was replaced with 55 mM iodoacetamide at 22℃ for 20 min in a dark room to alkylate the gels, followed by washing with 50% acetonitrile in 50 mM ammonium bicarbonate at 22℃ for 15 min and dehydrating with 100% ethanol for 5 min. The gel pieces were hydrated with 2.5 ng/µl of trypsin in 50 mM ammonium bicarbonate overnight at 37℃. Peptides were extracted with the following steps: twice with 25% acetonitrile with sonication in a water bath; 100% acetonitrile with sonication; supernatants were collected in a fresh vial, and dried using a vacuum centrifuge at 50℃, and resuspended in 50 µl of 2% acetonitrile and 0.1% trifluoroacetic acid.

LC-MS/MS analyses were performed using an EASY-nLC 1000 system and an Orbitrap-Fusion (Thermo Fisher Scientific, [Waltham,](https://cn.bing.com/search?q=waltham+massachusetts&filters=ufn%3a%22waltham+massachusetts%22+sid%3a%229ab6edbf-73c4-a900-b366-f55561bbe95f%22+catguid%3a%22619c033b-faf6-2849-e040-0fd3462d0282_acb135c0%22+segment%3a%22generic.carousel%22+gsexp%3a%22619c033b-faf6-2849-e040-0fd3462d0282_cmVsL29yZ2FuaXphdGlvbi5vcmdhbml6YXRpb24uaGVhZHF1YXJ0ZXJzfFRydWU.%22&FORM=SNAPST) MA, USA) according to a published protocol with modifications. One μg of peptides were loaded on an Acclaim-PepMap μ-precolumn (Thermo Scientific, 300 μm i.d. x 5 mm length, 5 μm particle size, 100 Å pore size), and equilibrated in 2% acetonitrile and 0.1% trifluoroacetic acid, for 8 min at 10 μl/min with an analytical column Acclaim PepMap RSLC (Thermo Fisher Scientific, 75 μm i.d. x 50 cm, 2 μm, 100 Å). Mobile phase A consisted of 0.1% formic acid, and mobile phase B consisted of 0.1% formic acid in 100% acetonitrile. Peptides were eluted at 300 nl/min by increasing the mobile phase B from 8% B to 28% over 107 min, then 90% B for 3 min, followed by a 15-min re-equilibration at 20% B. To avoid cross-contamination between samples, two washes of 30 min were run between samples. MS data were acquired with Xcalibur v3.0.63 (Thermo Fisher Scientific). Electrospray used a static Nanospray-Flex with a stainless-steel emitter OD 1/32’in positive mode at 2.2 kV (Thermo Scientific). MS survey scans from 350 to 1,550 m/z, with a 2x10^5^ ion count target, maximum injection time of 50 ms and resolution of 120,000 at 200 m/z, acquired in profile mode was performed in the Orbitrap analyzer. Data dependent mode selected the most abundant precursor ions possible in 2 s cycle time followed by 45 s exclusion and ions were isolated in the quadrupole with a 1.2 m/z window. MS/MS scanning was performed with rapid mode in the ion trap with ion count target of 1x10^4^ and maximum injection time of 45 ms and acquired in centroid mode. Precursor ions were fragmented with higher energy C-trap dissociation (HCD), normalized collision energy of 30% and fixed first mass of 120 m/z. Performance of the LC-MS was controlled by running HeLa lysates quality controls (Thermo Fisher Scientific) before and after the experiments. The raw files were analyzed using MaxQuant software v1.5.28 (MaxQuant, Martinsried, Germany) against the UniProtKB Human database. Peptide sequences were assigned to MS/MS spectra using the following parameters: cysteine carbamidomethylation as a fixed modification and methionine oxidations as variable modifications. The false discovery rate (FDR) was set to *Q* < 0.01 for both proteins and peptides with a minimum length of 7 amino acids and was determined by searching a reversed database.

**Parallel reaction monitoring analysis**

The differentially expressed proteins obtained from LC-MS/MS analysis were further validated by targeted proteomic analysis using parallel reaction monitoring (PRM) according to a published protocol with minor modifications.^1^ Briefly, the protein samples were prepared by the same method as used for LC-MS/MS analysis. PRM analyses were performed on a Q-Exactive mass spectrometer (Thermo Fisher Scientific). For ionization, 2,200 V and a 250-℃ capillary temperature were used. The reverse-phase liquid chromatography (RPLC) fractions of each sample were analyzed using an acquisition method. The combination of a full scan selected ion monitoring (SIM) event was followed by 14 PRM scans, which were triggered by an unscheduled inclusion list that contains the target precursor ions representing variant peptides. Normalized collision energy of 27 was used for fragmentation. A starting mass of m/z 150 was used for MS/MS scan. PRM data analysis was performed using Skyline software (MacCoss Lab, University of Washington, Seattle, WA, USA). Peptide settings: enzyme was set as Trypsin [KR/P]; max missed cleavage was set as 2; and the peptide length was set as 7-25. Transition settings: precursor charges were set as 2, 3; ion charges were set as 1, 2; ion types were set as b, y, p; the product ions were set as from ion 3 to last ion; and the ion match tolerance was set as 0.02Da.

1. Zhang B, Wang J, Wang X, et al. Proteogenomic characterization of human colon and rectal cancer. *Nature*. 2014;513(7518):382-387. doi:10.1038/nature13438
