# Supplementary figures and images for: Proteomic profiling of circulating plasma exosomes reveals novel biomarkers of Alzheimer’s disease
Source: Alzheimers Res Ther. 2022 Dec 5;14:181. doi: 10.1186/s13195-022-01133-1 (PMC9720984; doi:10.1186/s13195-022-01133-1)

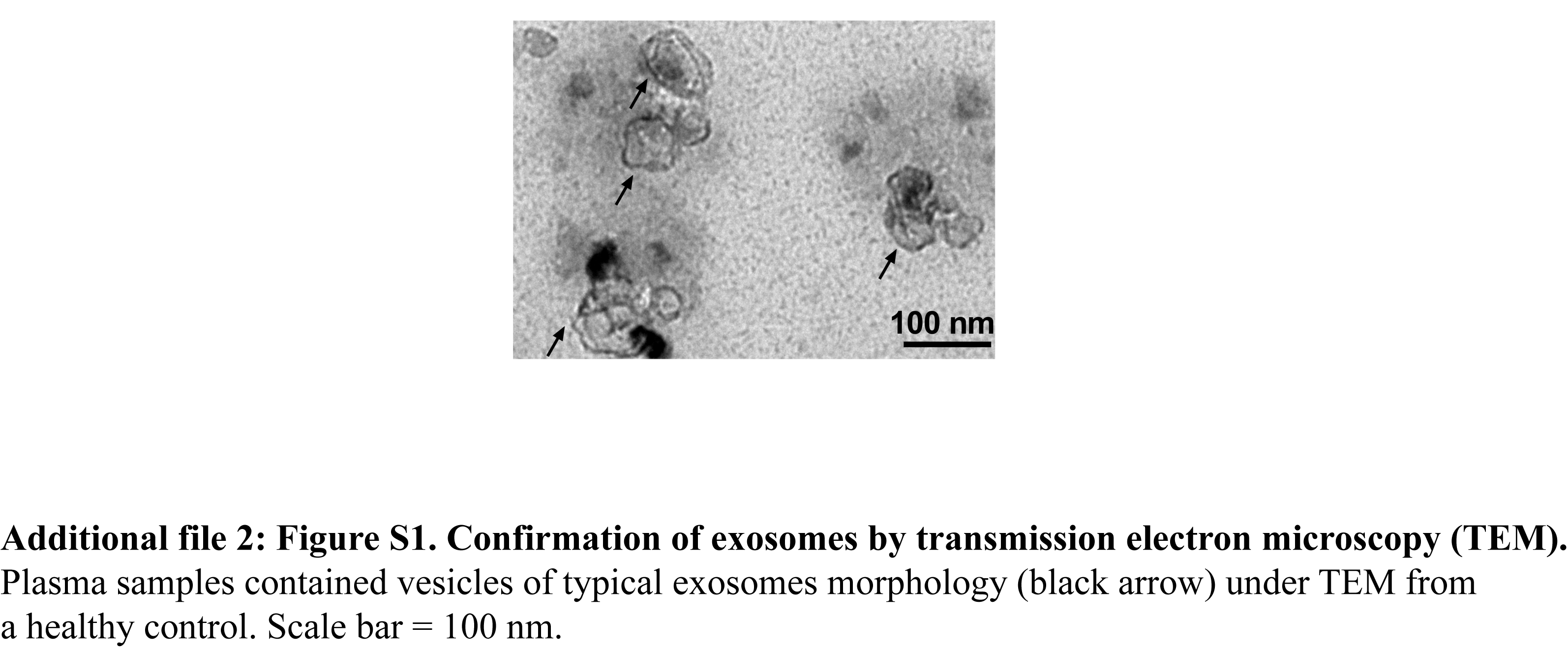

Supplement: Supplementary file 2 — Additional file 2: Figures S1. Confirmation of exosomes by transmission electron microscopy (TEM). [file 13195_2022_1133_MOESM2_ESM.tif]

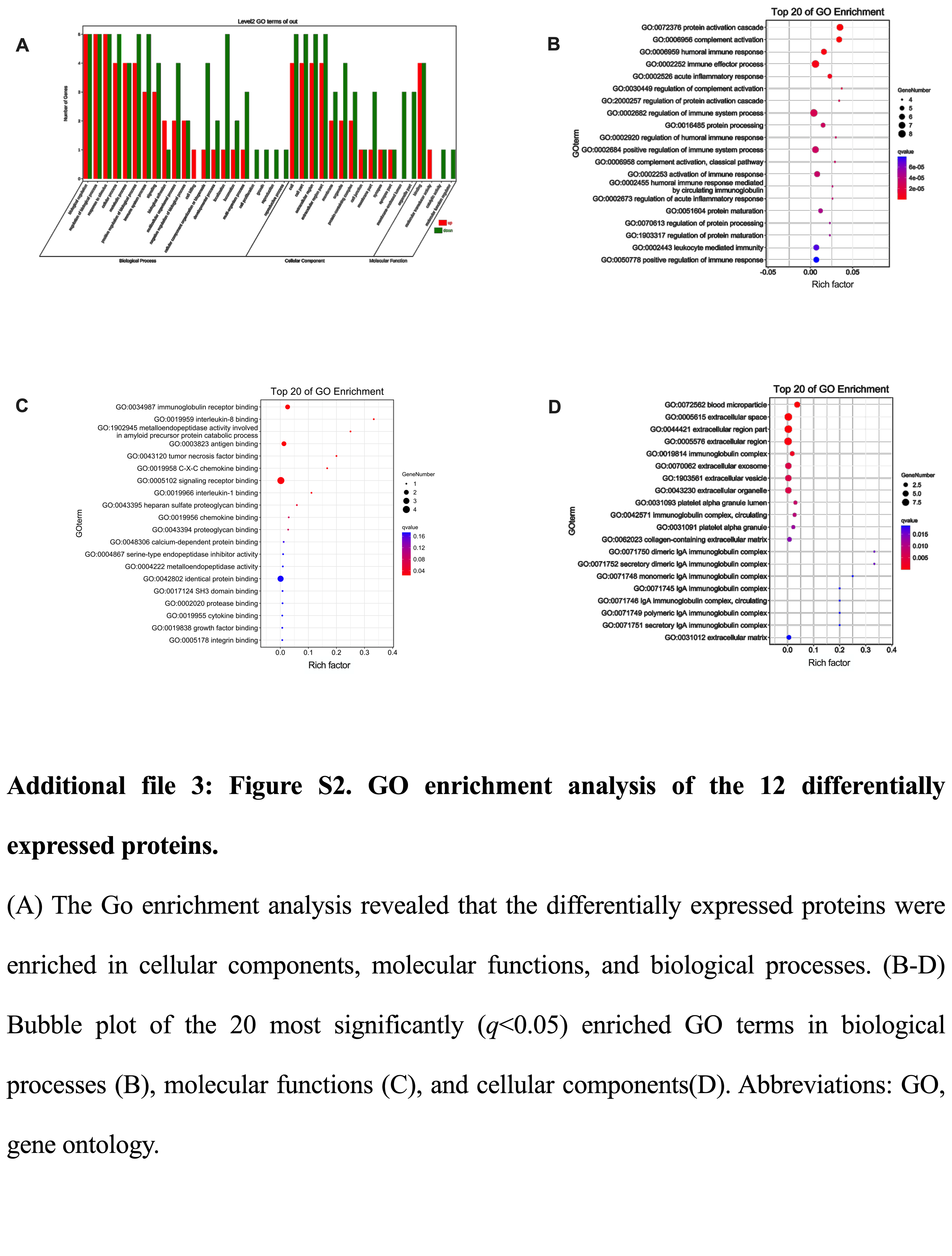

Supplement: Supplementary file 3 — Additional file 3: Figures S2. GO enrichment analysis of the 12 differentially expressed proteins. [file 13195_2022_1133_MOESM3_ESM.tiff]

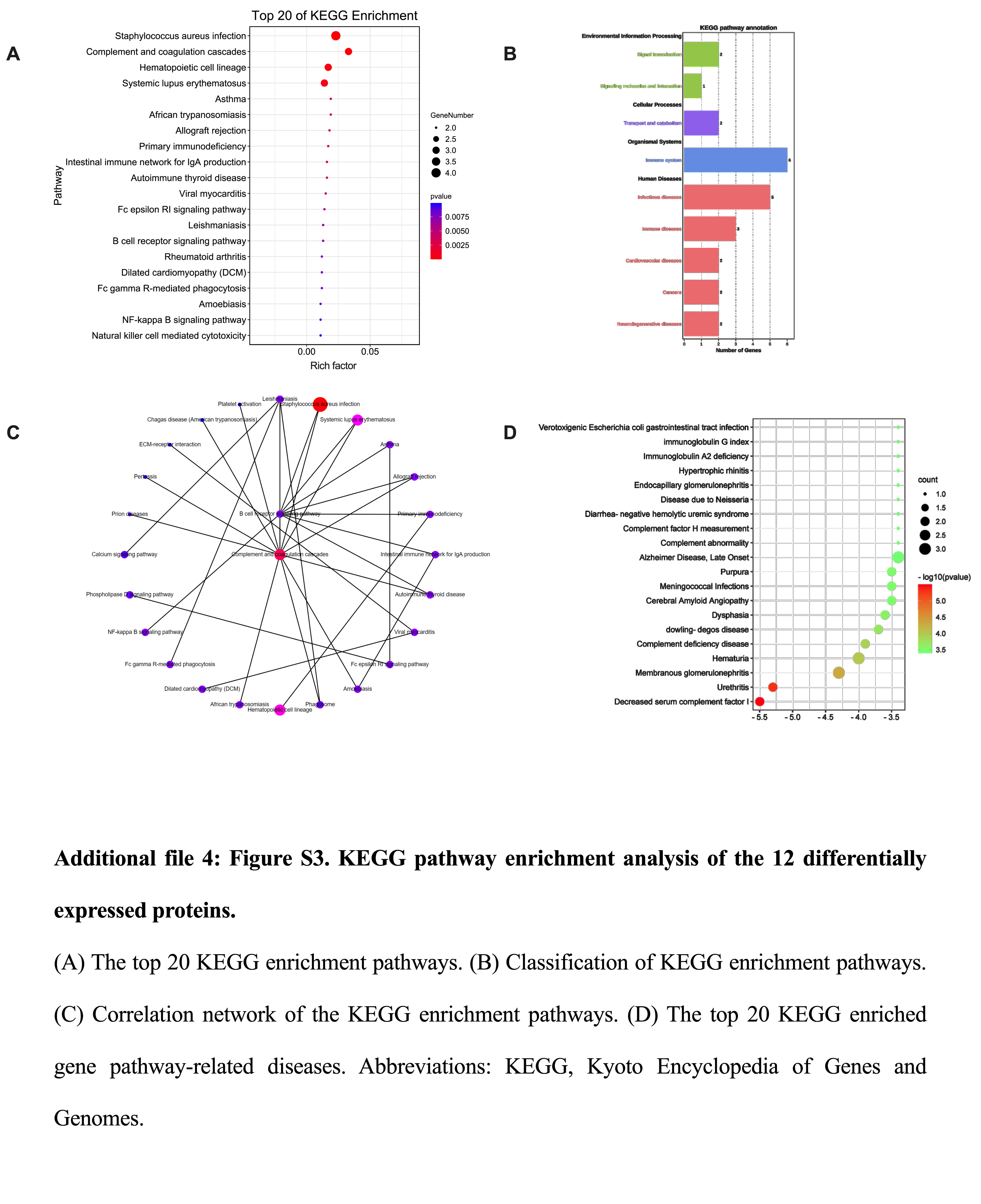

Supplement: Supplementary file 4 — Additional file 4: Figures S3. KEGG pathway enrichment analysis of the 12 differentially expressed proteins. [file 13195_2022_1133_MOESM4_ESM.tiff]
